# Accurate detection of *Neisseria gonorrhoeae* ciprofloxacin susceptibility directly from genital and extragenital clinical samples: towards genotype-guided antimicrobial therapy

**DOI:** 10.1093/jac/dkv432

**Published:** 2016-01-26

**Authors:** Marcus J. Pond, Catherine L. Hall, Victoria F. Miari, Michelle Cole, Ken G. Laing, Heena Jagatia, Emma Harding-Esch, Irene M. Monahan, Timothy Planche, Jason Hinds, Catherine A. Ison, Stephanie Chisholm, Philip D. Butcher, Syed Tariq Sadiq

**Affiliations:** 1 Institute for Infection and Immunity, St George's, University of London, London, UK; 2 Sexually Transmitted Bacteria Reference Unit, Public Health England, Colindale, London, UK; 3 Department of STI/HIV, Public Health England, Colindale, London, UK; 4 Medical Microbiology, South West London Pathology, St George's University Hospitals NHS Foundation Trust, London, UK; 5 Department of Genitourinary & HIV Medicine, St George's University Hospitals NHS Foundation Trust, London, UK

## Abstract

**Introduction:**

Increasing use of nucleic acid amplification tests (NAATs) as the primary means of diagnosing gonococcal infection has resulted in diminished availability of *Neisseria gonorrhoeae* antimicrobial susceptibility data. We conducted a prospective diagnostic assessment of a real-time PCR assay (NGSNP) enabling direct detection of gonococcal ciprofloxacin susceptibility from a range of clinical sample types.

**Methods:**

NGSNP, designed to discriminate an SNP associated with ciprofloxacin resistance within the *N. gonorrhoeae* genome, was validated using a characterized panel of geographically diverse isolates (*n* = 90) and evaluated to predict ciprofloxacin susceptibility directly on *N. gonorrhoeae*-positive NAAT lysates derived from genital (*n* = 174) and non-genital (*n* = 116) samples (*n* = 290), from 222 culture-confirmed clinical episodes of gonococcal infection.

**Results:**

NGSNP correctly genotyped all phenotypically susceptible (*n* = 49) and resistant (*n* = 41) panel isolates. Ciprofloxacin-resistant *N. gonorrhoeae* was responsible for infection in 29.7% (*n* = 66) of clinical episodes evaluated. Compared with phenotypic susceptibility testing, NGSNP demonstrated sensitivity and specificity of 95.8% (95% CI 91.5%–98.3%) and 100% (95% CI 94.7%–100%), respectively, for detecting ciprofloxacin-susceptible *N. gonorrhoeae*, with a positive predictive value of 100% (95% CI 97.7%–100%). Applied to urogenital (*n* = 164), rectal (*n* = 40) and pharyngeal samples alone (*n* = 30), positive predictive values were 100% (95% CI 96.8%–100%), 100% (95% CI 87.2%–100%) and 100% (95% CI 82.4%–100%), respectively.

**Conclusions:**

Genotypic prediction of *N. gonorrhoeae* ciprofloxacin susceptibility directly from clinical samples was highly accurate and, in the absence of culture, will facilitate use of tailored therapy for gonococcal infection, sparing use of current empirical treatment regimens and enhancing acquisition of susceptibility data for surveillance.

## Introduction

*Neisseria gonorrhoeae* infection is frequently treated empirically at the point of care (PoC), based on clinical presentation alongside findings from microscopy of Gram-stained genital swabs.^1^ Efficacy of antimicrobial therapy is threatened by the development of successive antimicrobial resistance (AMR) in response to antibiotic classes used over time,^2^ resulting in potentially empirically untreatable gonorrhoea.^3^ In addition, nucleic acid amplification tests (NAATs) have largely replaced culture as the primary laboratory method of gonorrhoea diagnosis,^4,5^ resulting in a decline in availability of antibiotic susceptibility data to guide prescribing.^6^

These challenges have instigated the development of the WHO Global action plan to control the spread and impact of AMR in *N. gonorrhoeae*,^7^ recognizing the need for molecular methods for monitoring and detecting AMR. Fluoroquinolones may represent a favourable group of antimicrobials to which molecular AMR detection may be applied due to the relative genetic simplicity of resistance, mediated predominantly through SNPs within genes coding for the GyrA subunit of DNA gyrase and ParC subunit of topoisomerase IV.^8^

Deployment of genotypic methods for the detection of AMR in *N. gonorrhoeae* is potentially challenged by cross-reaction with commensal *Neisseria* species at extragenital sites, detrimentally affecting assay specificity.^9^ The aim of this study was to ascertain the diagnostic performance of a real-time PCR assay (NGSNP), enabling genotypic prediction of ciprofloxacin-susceptible or -resistant *N. gonorrhoeae* directly on clinical samples taken from diverse sites. NGSNP functionality was verified using a geographically diverse gonococcal isolate panel and its capacity to genotypically predict gonococcal fluoroquinolone susceptibility, directly from residual NAAT samples, was evaluated through comparison with routine antimicrobial susceptibility testing (AST).

## Materials and methods

### NGSNP real-time PCR assay

NGSNP was designed to discriminate an SNP occurring within the *gyrA* gene of the gonococcal genome. This SNP was identified because of its well-characterized association with phenotypic resistance to ciprofloxacin.^8^

Oligonucleotides were designed using Primer Express 3.0 software (Life Technologies, Foster City, CA, USA). PCR primers were designed to amplify a 77 bp region (nucleotide coordinates 620 887–620 954) of *gyrA* (locus tag: NGO0629) of *N. gonorrhoeae* strain FA1090 (nucleotide accession NC_002946.2). TaqMan hydrolysis probes (Life Technologies, Paisley, UK) were designed to discriminate a cytosine to thymine SNP at nucleotide position 620 919 (see Table S1, available as Supplementary data at *JAC* Online).

Real-time PCR reactions were performed in a final volume of 20 μL, consisting of 10 μL of SsoFast™ Probes Supermix (Bio-Rad Laboratories, Hemel Hempstead, UK) and 5 μL of extracted clinical sample or 0.1 ng of bacterial genomic DNA. Final reaction primer and probe concentrations are detailed in the Supplementary Methods. NGSNP reactions were performed in duplicate using a CFX96 (Bio-Rad) real-time thermal cycler. PCR thermocycling conditions were 95°C for 5 min; 45 cycles of 95°C for 15 s; 60°C for 60 s. Real-time fluorescence detection was performed during the 60°C annealing/extension step of each cycle.

Post-PCR analysis of reaction fluorescence was performed using Bio-Rad CFX Manager software version 3.0. Thresholds for reaction positivity were set at 2000 relative fluorescence units to background for both FAM (510–530 nm) and VIC (560–580 nm) channels. Discrimination of genotypes was conducted with horizontal (WT) and vertical (SNP) alleles set to a Cq of 45. Three nucleic acid controls for real-time PCR runs were used at a normalized concentration of 0.1 ng/mL each. These controls consisted of nucleic acid from: strain FA1090 (WT with respect to antibiotic resistance); strain SGUL_239 (a ciprofloxacin-resistant local isolate possessing the base change at nucleotide position 620919 and having a ciprofloxacin MIC of 32 mg/L); and an equimolar mixture of nucleic acid from both these strains. Testing clinical and validation panels; SNP detection assays were performed blinded to phenotypic susceptibility results.

### Nationally representative ciprofloxacin-resistant N. gonorrhoeae panel

Ciprofloxacin-resistant isolates (MIC >0.5 mg/L) with an associated ST using *N. gonorrhoeae* multiantigen sequence typing (NG-MAST) archived between 2000–12 at the Sexually Transmitted Bacteria Reference Unit (STBRU), PHE, as part of the UK Gonococcal Resistance to Antimicrobials Surveillance Programme (GRASP) and European Gonococcal Antimicrobial Surveillance Programme collections were identified to create a porB/tbpB phylogenetically diverse panel of isolates, circulating within the UK and Europe (see the Supplementary Methods for culture methodology).

### Locally representative N. gonorrhoeae panel

An additional panel of isolates was identified to provide indicative performance within our local setting. Sixty-seven consecutively collected isolates from St George's University Hospitals NHS Foundation Trust (SGH), submitted and characterized as part of 2013 GRASP,^10^ were retrieved from STBRU. NG-MAST^11^ was performed on all isolates (see the Supplementary Methods for culture and NG-MAST methodology).

### Clinical specimens

This prospective diagnostic assessment was performed anonymized using residual lysates derived from routine NAAT diagnosis that only allowed for sample site and gender details to be collected. No details on identity, sexual behaviour, age, ethnicity or other demographics were available to the investigators and local research governance advised that a formal process of ethics committee approval was not required for this study.

Clinical episodes of culture-positive *N. gonorrhoeae* infection at any anatomical site from the Courtyard Genitourinary Medicine Clinic of SGH in London, UK, over a 9 month period between 15 October 2012 and 18 July 2013, were identified. Residual NAAT samples derived from these episodes consisting of lysed specimens derived from the BD ProbeTec GC Qx Amplified DNA Assay, performed on the Viper platform (Becton Dickinson, Oxford, UK), were stored for 1 week at 4°C for potential inclusion in the study.

Samples were eligible for inclusion if they came from the following routinely encountered sites: vaginal, cervical, pharyngeal and rectal swabs and urine specimens. Repeat clinical episodes from identical patients were included if separated by ≥8 weeks from previous episodes. Following meeting the inclusion criteria, the samples were anonymized so that the only available data were anatomical site sampled and antimicrobial susceptibility profile of the *N. gonorrhoeae* cultured during the same clinical attendance.

Antimicrobial susceptibility data for each clinically positive *N. gonorrhoeae* episode were obtained as part of routine diagnostic testing of patients by the Department of Medical Microbiology, SGH. Susceptibility data were recorded for the following antimicrobials: cefalexin, cefotaxime, ciprofloxacin, nalidixic acid, penicillin, spectinomycin and tetracycline. Testing was performed in accordance with BSAC methodology, with antimicrobial susceptibility reported as susceptible, intermediate or resistant depending on the disc diffusion zone diameter.^12^

Gonococcal nucleic acids were extracted from lysed NAAT samples by centrifugation of 500 μL of residual sample at 20 000 **g** for 5 min. Following centrifugation, 250 μL of supernatant was removed and the remaining liquid and pellet were used for total DNA extraction using the PowerLyzer™ PowerSoil^®^ DNA Isolation Kit according to the manufacturer's instructions (MoBio, Carlsbad, CA, USA). DNA was eluted in a final volume of 100 μL of nuclease-free water and stored at −20°C until addition to real-time PCR reactions.

### Statistical analysis

Results of NGSNP testing directly on residual *N.* *gonorrhoeae*-positive NAAT lysates was compared with findings of routine culture-based AST. A sample size of 220 prospective clinical episodes was determined to be required in order to assess the performance of NGSNP with 98% accuracy at a 95% CI of 95%–100% (β = 80%, α = 0.05). CIs were calculated using a Wilson score, assuming a binomial distribution, with MedCalc statistical software (MedCalc, Ostend, Belgium). χ^2^ tests were used to detect differences in the failure rate of NGSNP and prevalence of ciprofloxacin resistance at different sampling sites. Statistical analysis was performed using SPSS version 21 and MedCalc statistical software.

## Results

Assay functionality was initially demonstrated by testing NGSNP with genomic DNA extracted from a geographically diverse panel of 23 ciprofloxacin-resistant *N. gonorrhoeae* isolates representing 22 NG-MAST types (see Table S2). The median ciprofloxacin MIC for these isolates was 7 mg/L (IQR 4–32 mg/L). All isolates included within this panel were successfully genotyped as ‘resistant’ by the generation of a fluorescence signal within the SNP detection (VIC) channel of the real-time PCR reaction. The capacity of NGSNP was further verified using a panel of local *N. gonorrhoeae* isolates, originally collected at SGH. NGSNP reactions performed using genomic DNA extracted from these isolates produced an SNP signal for all 18 (27%) demonstrated to be phenotypically resistant to ciprofloxacin [median MIC of 16 mg/L (IQR 8–16 mg/L)] and a WT signal for all remaining 49 phenotypically susceptible isolates. NG-MAST types were available for all 67 isolates and 40 different STs were observed (see Table S3).

Following successful validation, the diagnostic performance of NGSNP was assessed using 290 NAAT specimens from 222 (56 female and 166 male) clinical episodes of culture-positive gonorrhoea that met the inclusion criteria during the study period (Tables 1 and 2). Two female patients were sampled on two separate clinical episodes. Three male patients were sampled on two separate clinical episodes and a single male patient provided samples attributable to four episodes. Repeat clinical episodes were separated by a minimum of 8 weeks and a maximum of 8 months.

**Table 1.** Distribution of NGSNP sample types and site used for corresponding AST: female samples (*n* = 69)

	‘Genital’				‘Non-genital’		
NGSNP sample site [count]^a^	cervical [33]	vaginal [20 (3)]			pharyngeal [12 (2)]		rectal [4]
Susceptibility testing site	cervical	cervical	urethral	vaginal	pharyngeal	cervical	rectal
Count^b^	33 (5)	17 (4)	2 (1)	1	10 (4)	2	4

^a^Numbers in parentheses represent the number of NGSNP assays that failed to yield a result.

^b^Numbers in parentheses represent the number of fluoroquinolone-resistant cultures of *N. gonorrhoeae*.

**Table 2.** Distribution of NGSNP sample types and site used for corresponding AST: male samples (*n* = 221)

	‘Genital’		‘Non-genital’					
NGSNP sample site [count]^a^	urine [121 (6)]		pharyngeal [44 (9)]			rectal [56 (8)]		
Susceptibility testing site	urethral	pharyngeal	urethral	pharyngeal	rectal	urethral	pharyngeal	rectal
Count^b^	120 (39)	1	9 (2)	24 (8)	11 (3)	18 (6)	2 (2)	36 (12)

^a^Numbers in parentheses represent the number of NGSNP assays that failed to yield a result.

^b^Numbers in parentheses represent the number of fluoroquinolone-resistant cultures of *N. gonorrhoeae*.

One hundred and seventy-four samples included in this study were defined as genital (cervical, vaginal and urethral samples in women and urethral swab and urine in men) and 116 samples were defined as non-genital (throat or rectal swabs in males or females). Some 99.4% of genital and 63.8% of non-genital samples specimens had a positive *N. gonorrhoeae* culture from the same sample site (see Tables 1 and 2). For this analysis, cervical, vaginal and urethral samples in women and urethral swab and urine samples in men were assumed to constitute the same genital site.

Of the clinical episodes, 28.8% (64/222) were attributable to the presence of ciprofloxacin-resistant *N. gonorrhoeae* in at least one anatomical site (Table 3), with a higher proportion observed in men compared with women [32.5% (54/166) versus 17.9% (10/56); χ^2^ = 4.393, *P* = 0.04] and in non-genital sites compared with genital sites [35.4% (35/99) versus 23.6% (29/123), respectively; χ^2^ = 3.707, *P* = 0.054].

**Table 3.** Frequency of infected anatomical sites at patient's clinical episode

	Sample site						
	genital only	pharyngeal only	rectal only	genital and pharyngeal	genital and rectal	genital, rectal and pharyngeal	rectal and pharyngeal
Female episodes (*n* = 56)	42 (6)	1	1	9 (2)	0	3 (2)	0
Male episodes (*n* = 166)	81 (23^a^)	12 (3)	14 (6)	11 (4)	19 (9)^b^	11 (3)	18 (6)

Numbers in parentheses represent the number of cases attributable to a fluoroquinolone-resistant isolate of *N. gonorrhoeae*.

^a^A single male patient was infected with two strains of *N. gonorrhoeae*; this mixed infection was identified as one strain susceptible to tetracycline and one strain resistant.

^b^All patients with ciprofloxacin-resistant gonorrhoea had concordant phenotypic results at different sites except for three men with genital and rectal cultures. In these men, there were two cases of rectal resistance and urethral susceptibility and one case of urethral resistance and rectal susceptibility.

Multisite *N. gonorrhoeae* infection was observed in 71 (59 male and 12 female) clinical episodes (Table 3). In three of these episodes (4.2%), all of which were in men, infection was attributable to isolates with discordant ciprofloxacin susceptibility profiles at different anatomical sites. Two patients possessed resistant isolates in genital cultures whereas the remaining patient was infected with a resistant isolate in their rectal culture; the remaining infected sites in these patients were attributable to ciprofloxacin-susceptible *N. gonorrhoeae* infection.

Total DNA was extracted from all residual sample lysates included in the study and tested using NGSNP, yielding a result in 90% (262/290) of samples (Tables 1 and 2). Failure (no amplification) of NGSNP was associated with non-genital sites compared with genital samples (16.4% versus 5.2%; χ^2^ = 10.021, *P* = 0.002) and a greater proportion of assay failures occurred in samples in which culture results were derived from different sample sites (34.9% versus 5.3%; χ^2^ = 36.83, *P* < 0.001).

Prior to determining the diagnostic performance of NGSNP, 28 samples were excluded from the final analysis due to assay failure. Forty-three of the 290 samples were excluded from the final analysis of NGSNP accuracy as susceptibility testing data for these samples were not available from a matching anatomical site (see Tables 1 and 2).

Where NAAT and phenotypic susceptibility sampling were performed at the same site (Table 4), NGSNP demonstrated sensitivity and specificity for detecting ciprofloxacin susceptibility of 95.8% (95% CI 91.5%–98.3%) and 100% (95% CI 94.7%–100%), respectively. Therefore, absence of the SNP demonstrated 100% (95% CI 97.7%–100%) positive predictive value to predict ciprofloxacin susceptibility of all culture-matched genital, pharyngeal and rectal samples (Table 4).

**Table 4.** NGSNP assay performance for detection and prediction of ciprofloxacin susceptibility from genital and non-genital samples compared with phenotypic susceptibility test from the same anatomical site

Episode type	Sensitivity (%)	Specificity (%)	Positive predictive value (%)^a^	Negative predictive value (%)^b^
Overall (*n* = 234)	95.8 (91.5–98.3)	100 (94.7–100)	100 (97.7–100)	90.7 (81.7–96.2)
female and male cases of urogenital infection (*n* = 164)	95.8 (90.4–98.6)	100 (92.3–100)	100 (96.8–100)	90.2 (78.6–96.7)
female cases (*n* = 50)	97.5 (86.8–99.9)	100 (69.2–100)	100 (91.0–100)	90.9 (58.7–99.8)
male cases (*n* = 114)	94.9 (87.4–98.6)	100 (90.3–100)	100 (95.1–100)	89.7 (76.3–97.2)
female and male cases of non-genital infection (*n* = 70)	95.8 (85.8–99.5)	100 (84.6–100)	100 (92.3–100)	91.7 (73.0–99.0)
female and male cases of pharyngeal infection (*n* = 30)	100 (82.4–100)	100 (71.5–100)	100 (82.4–100)	100 (71.5–100)
female and male cases of rectal infection (*n* = 40)	93.1 (77.2–99.2)	100 (71.5–100)	100 (87.2–100)	84.6 (54.6–98.1)

Numbers in parentheses are the 95% CIs. Performance was evaluated on those in which the assay was successful (see Tables 1 and 2 for total number of cases and tests). Test positivity is defined as the absence of the serine 91 to phenylalanine mutation.

^a^This value represents the predictive value of the absence of the mutation for ciprofloxacin susceptibility.

^b^This value represents the predictive value of the presence of the mutation for ciprofloxacin resistance.

## Discussion

NAATs have surpassed bacteriological culture as the primary means of diagnosing *N. gonorrhoeae* infection,^4,5^ frequently resulting in clinical management of gonorrhoea in the absence of available antibiotic susceptibility data.^6^ In order to address this deficiency in clinical practice, we developed and prospectively evaluated a real-time PCR assay enabling genotypic detection of gonococcal susceptibility to ciprofloxacin from residual NAAT samples. This approach facilitated accurate prediction of gonococcal susceptibility to ciprofloxacin directly from residual genital and non-genital patient samples. In patients diagnosed exclusively by NAATs and recalled for treatment, prompt testing of *N.* *gonorrhoeae*-positive samples using NGSNP would enable the ciprofloxacin susceptibility status to be provided on the patient's return, aiding clinical management.

Use of a single 500 mg dose of ciprofloxacin has not been recommended for empirically treating uncomplicated gonorrhoea in the UK since 2002, when resistance rates increased beyond 5%, rendering it unsuitable.^13^ However, as of 2013, >70% of *N. gonorrhoeae* isolates analysed by GRASP were susceptible to ciprofloxacin,^10^ suggesting availability of susceptibility data would allow almost 20 000 of ∼29 000 cases of gonorrhoea diagnosed in England and Wales in 2013 to include ciprofloxacin as a viable treatment option.

We previously proposed that integration of genotypic markers of AMR into NAATs and PoC tests may enable older antibiotics to be used as effective treatments at diagnosis once again.^14^ The highly specific nature of NGSNP and the genotypic assay underpinning it has the potential for such integration, possibly with existing NAATs.^15^ This would enable giving susceptibility-guided ciprofloxacin therapy at PoC, either as monotherapy or as an adjunctive component of combination therapy. Such personalized diagnostics could serve to preserve the use of newer and future empirical antigonococcal antibiotics such as extended-spectrum cephalosporins, gentamicin^16^ and solithromycin.^17^

Worldwide, the proportion of *N. gonorrhoeae* isolates identified as ciprofloxacin susceptible from regional and national surveillance programmes varies by geographic location. These estimates include 29%–100% in Africa,^18^ 52.9% in Europe,^19^ 74.5% in Russia,^20^ 86.5% in the USA,^21^ 57.9% in South America and the Caribbean,^22^ 0%–62% of isolates in South-East Asia Region^23^ and 1%–100% in the Western Pacific,^24^ indicating the potential global utility of such an approach.

Validation of NGSNP performance on phylogenetically diverse isolates was a clear strength of the study, indicating applicability of the selected *gyrA* marker of resistance beyond our local population. We have conducted a review of published studies, which suggests that a residue change at codon 96 of GyrA occurs in 96% of ciprofloxacin-resistant strains of *N. gonorrhoeae* (C. L. Hall, M. J. Pond, E. Harding-Esch, S. T. Sadiq, unpublished data), though we also note intermediate susceptibility to ciprofloxacin may occur in isolates with alterations at codon 95 of GyrA alone.^25^ In addition to SNPs within *gyrA*, resistant isolates may demonstrate additional genomic changes that give rise to residue changes at codons 86 aspartate, 87 serine, 88 serine and 91 glutamate of ParC.^26^ Inclusion of these genomic markers in a future assay design may improve assay sensitivity for the detection of susceptible gonococcal infections. The presence of other independent markers^27,28^ of ciprofloxacin resistance noted in other bacterial species demonstrates the need for continuing phenotypic/genotypic surveillance^29^ to inform genotypic susceptibility assays.

This study highlighted an additional complication of utilizing NAATs for *N. gonorrhoeae* infection, namely the reduction in assay success observed when diagnosing non-genital infection.^9^ NGSNP assay failure occurred more frequently in extragenital NAAT-positive samples. At the time of this study, non-genital NAAT-positive specimens were not confirmed with a secondary NAAT assay if patients were culture positive at an additional site.

Interestingly, among non-genital samples excluded from analysis due to susceptibility testing data derived from a non-matching anatomical site, a decline in NGSNP test performance was observed. Although this finding may be attributable to NAAT false positivity,^9^ the possibility of infection with *N. gonorrhoeae* clones of discordant antimicrobial susceptibility at each anatomical site cannot be excluded.

This hypothesis is strengthened by the observation that three men were indeed infected with isolates possessing different antimicrobial susceptibility profiles at separate anatomical sites simultaneously. Furthermore, a single case of mixed gonococcal infection within a case of male urethral infection (see Table 3) was observed, though both isolates were resistant to ciprofloxacin and did not affect the assay under analysis.

These observations suggest a requirement for multisite testing when deploying genotypic resistance susceptibility testing for gonorrhoea and that present culture techniques may underdetect mixed *N. gonorrhoeae* infection.^30,31^ Accurately quantifying rates at which mixed gonococcal infection occurs within anatomical sites and within patients will improve both molecular and conventional methods of susceptibility testing.

In conclusion, NGSNP affords highly accurate genotypic prediction of gonococcal ciprofloxacin from routine clinical samples of different anatomical sites, demonstrating that provision of susceptibility data in the absence of culture is feasible. This approach may serve to enhance current empirical treatment regimens and spare the use of newer antibiotics, providing an additional pragmatic approach to address the spread of MDR gonorrhoea.

## Funding

This work was supported by the UK Clinical Research Collaboration (Medical Research Council) Translation Infection Research Initiative Consortium (grant number G0901608).

## Transparency declarations

None to declare.

### Author contributions

M. J. P. and S. T. S. jointly conceived the study. S. T. S., M. J. P., P. D. B., J. H., K. G. L., V. F. M., H. J., C. L. H. and E. H.-E. designed and planned the study and/or performed laboratory work. C. A. I., S. C. and M. C. characterized strains and contributed to the study design. I. M. M. and T. P. supported initial validation. M. J. P. and S. T. S. drafted the manuscript and all authors contributed to the manuscript.

## Supplementary data

Additional Methods and Tables S1 to S3 are available as Supplementary data at *JAC* Online (http://jac.oxfordjournals.org/).

Supplementary Data
