# Supplementary material for: Accurate detection of Neisseria gonorrhoeae ciprofloxacin susceptibility directly from genital and extragenital clinical samples: towards genotype-guided antimicrobial therapy
Source: J Antimicrob Chemother. 2016 Jan 26;71(4):897–902. doi: 10.1093/jac/dkv432 (PMC4790619; doi:10.1093/jac/dkv432)
Supplement: Supplementary Data [file supp_71_4_897__index.html]

Accurate detection of Neisseria gonorrhoeae ciprofloxacin susceptibility directly from genital and extragenital clinical samples: towards genotype-guided antimicrobial therapy — Accurate detection of Neisseria gonorrhoeae ciprofloxacin susceptibility directly from genital and extragenital clinical samples: towards genotype-guided antimicrobial therapy — Supplementary Data 

# Accurate detection of *Neisseria gonorrhoeae* ciprofloxacin susceptibility directly from genital and extragenital clinical samples: towards genotype-guided antimicrobial therapy

## Supplementary Data

Supplementary Data

- Supplementary Data - Docx file
