# Supplementary material for: Accurate detection of Neisseria gonorrhoeae ciprofloxacin susceptibility directly from genital and extragenital clinical samples: towards genotype-guided antimicrobial therapy
Source: J Antimicrob Chemother. 2016 Jan 26;71(4):897–902. doi: 10.1093/jac/dkv432 (PMC4790619; doi:10.1093/jac/dkv432)
Supplement: Supplementary Data [file supp_dkv432_dkv432supp.docx]

**Supplementary data**

**Supplementary methods**

***N. gonorrhoeae* culture techniques**

All isolates were retrieved from frozen growth in glycerol stocks at the Sexually Transmitted Bacteria Reference Unit of Public Health England, Colindale, London, United Kingdom (STBRU) and inoculated onto chocolate agar (Oxoid, Basingstoke, United Kingdom) and incubated for 24-48 h in 5% carbon dioxide at 35 ºC. and resistance was defined as an MIC of >0.5 mg/L. A 10 µL loopful of bacterial growth was used for genomic DNA extraction using a QIAmp DNA Mini Kit (QIAGEN, Crawley, United Kingdom) according to manufacturer’s instructions for extraction of Gram-negative bacteria. Extracted genomic DNA was normalised to a concentration of 1 ng/µL and stored at -20 ºC until required.

**NG-MAST methodology**, PCR reactions were performed in a final reaction volume of 50 µL using Q5® High-Fidelity 2X Master Mix (New England Biolabs, Ipswich, MA), 2 µl extracted DNA and 250 nM of each primer. Primers for *porB* and *tbpB* amplification and sequencing have been described previously.^12^ Cycling conditions for *tbpB* amplification was 30 s at 98 °C; 40 cycles of 98 °C for 10 s; 71 °C for 30 s; 72 °C for 30 s and final extension 72 °C for 2 min. Cycling conditions for *porB* amplification were the same except for an annealing temperature at 57 °C. Amplified regions of *porB* and *tbpB* were sequenced (Source BioScience, Nottingham, United Kingdom) and alleles at *porB* and *tbpB* and NG-MAST STs assigned at the NG-MAST website ([www.ng-mast.net](http://www.ng-mast.net)).

**Table S1. Oligonucleotide primers and probes used**

| **Oligonucleotide** | **Sequence 5’ to 3’** | **Nucleotide position within**  **NC_002946.2** | **Final reaction concentration** |
| --- | --- | --- | --- |
| NG_gyrA_F | GCGACGTCATCGGTAAATACC | 620934 to 620954 | 300 nM |
| NG_gyrA_R | CGCCATACGGACGATGGT | 620887 to 620904 (plus strand) | 900 nM |
| NG_gyrA_SNP_PR | VIC-CCCCCACGGCGATTTCGCAGTT- BHQ1 | 620911 to 620932 | 200 nM |
| NG_gyrA_WT_PR | FAM-CCCCACGGCGATTCCGCAGT- BHQ1 | 620912 to 620931 | 200 nM |

**Table S2. Isolates used as ciprofloxacin-resistant isolate panel (*n*=24).** Sequence type derived using NG-MAST [32] SNP=presence of C>T mutation at position 620919 of *gyrA,* Ciprofloxacin resistance defined as an MIC of>0.5 mg/L

| **Sequence Type** | **Ciprofloxacin MIC (mg/L)** | **NGSNP Result** | **Region ST reported** |
| --- | --- | --- | --- |
| 1407 | >32 | SNP | UK and Non-UK |
| 3158 | 24 | SNP | UK and Non-UK |
| 1634 | >32 | SNP | UK |
| 4822 | 16 | SNP | UK and Non-UK |
| 1479 | 1 | SNP | UK and Non-UK |
| 753 | 3 | SNP | UK |
| 5584 | 6 | SNP | UK |
| 225 | 3 | SNP | UK and Non-UK |
| 147 | >32 | SNP | UK and Non-UK |
| 2212 | 4 | SNP | UK and Non-UK |
| 3520 | 4 | SNP | UK |
| 1440 | 8 | SNP | UK |
| 1407 | 4 | SNP | UK and Non-UK |
| 3302 | 4 | SNP | UK and Non-UK |
| 10032 | 4 | SNP | UK |
| 3149 | 32 | SNP | UK and Non-UK |
| 3405 | >32 | SNP | UK |
| 437 | 32 | SNP | UK and Non-UK |
| 5350 | 32 | SNP | UK |
| 2400 | 12 | SNP | Non-UK |
| 1582 | 4 | SNP | UK and Non-UK |
| 3387 | >32 | SNP | UK and Non-UK |
| 1782 | 6 | SNP | UK |
| 1782 | 6 | SNP | UK |

**Table S3. Isolates used as locally representative isolate panel n=66.** Sequence type derived using NG-MAST [32]. SNP/WT=presence/absence of C>T mutation at position 620919 of *gyrA.* Ciprofloxacin resistance defined as an MIC of>0.5 mg/L

| **Sequence Type** | **Ciprofloxacin MIC (mg/L)** | **NGSNP Result** |
| --- | --- | --- |
| 2400 | 16 | SNP |
| 51 | ≤0.030 | WT |
| 292 | ≤0.030 | WT |
| 51 | ≤0.030 | WT |
| 51 | ≤0.030 | WT |
| 11640 | 16 | SNP |
| 21 | ≤0.030 | WT |
| 8989 | >16 | SNP |
| 51 | ≤0.030 | WT |
| 2400 | 16 | SNP |
| 4995 | 8 | SNP |
| 6837 | ≤0.030 | WT |
| 1978 | >16 | SNP |
| 1407 | 16 | SNP |
| 7651 | ≤0.030 | WT |
| 10149 | 8 | SNP |
| 995 | ≤0.030 | WT |
| 4016 | ≤0.030 | WT |
| 11641 | 16 | SNP |
| 10419 | ≤0.030 | WT |
| 4275 | 16 | SNP |
| 21 | ≤0.030 | WT |
| 1780 | ≤0.030 | WT |
| 2992 | ≤0.030 | WT |
| 1978 | >16 | SNP |
| 292 | ≤0.030 | WT |
| 11619 | ≤0.030 | WT |
| 6360 | 16 | SNP |
| 11641 | 16 | SNP |
| 6339 | ≤0.030 | WT |
| 470 | ≤0.030 | WT |
| 3369 | ≤0.030 | WT |
| 51 | ≤0.030 | WT |
| 5119 | ≤0.030 | WT |
| 2992 | ≤0.030 | WT |
| NEW POR ALLE | ≤0.030 | WT |
| 51 | ≤0.030 | WT |
| 292 | ≤0.030 | WT |
| 2992 | ≤0.030 | WT |
| 8435 | ≤0.030 | WT |
| 9705 | ≤0.030 | WT |
| 391 | ≤0.030 | WT |
| 51 | ≤0.030 | WT |
| 9901 | ≤0.030 | WT |
| 10149 | 8 | SNP |
| 10149 | 8 | SNP |
| 8919 | ≤0.030 | WT |
| 273 | ≤0.030 | WT |
| 26 | ≤0.030 | WT |
| NEW POR ALLE | ≤0.030 | WT |
| 292 | ≤0.030 | WT |
| NEW ST | 4 | SNP |
| 2992 | ≤0.030 | WT |
| 2992 | ≤0.030 | WT |
| 21 | ≤0.030 | WT |
| 1034 | ≤0.030 | WT |
| 21 | ≤0.030 | WT |
| 25 | ≤0.030 | WT |
| 26 | ≤0.030 | WT |
| 391 | ≤0.030 | WT |
| 51 | ≤0.030 | WT |
| 8465 | ≤0.030 | WT |
| 9808 | 16 | SNP |
| 2992 | ≤0.030 | WT |
| 26 | ≤0.030 | WT |
| 8987 | 16 | SNP |
